# Structural insights into the Omicron spike trimer: tackling the challenges of continuously evolving SARS-CoV-2 variants

**DOI:** 10.1038/s41392-022-01179-5

**Published:** 2022-09-16

**Authors:** Junxian Ou, Jianguo Wu, Qiwei Zhang

**Affiliations:** Guangdong Provincial Key Laboratory of Virology, Institute of Medical Microbiology, Jinan University, 510632 Guangzhou, China

**Keywords:** Microbiology, Diseases

The recent studies published in *Science and Cell Research* investigated the structures and biochemical mechanism of the SARS-CoV-2 Omicron BA.1 and BA.2 spike trimer with ACE2 as well as an effective antibody JMB2002 against BA.1 and BA.2 spike, which provide new insights for the global development of broad-spectrum anti-SARS-CoV-2 antibodies and vaccines.^1,2^

**Fig. 1:**
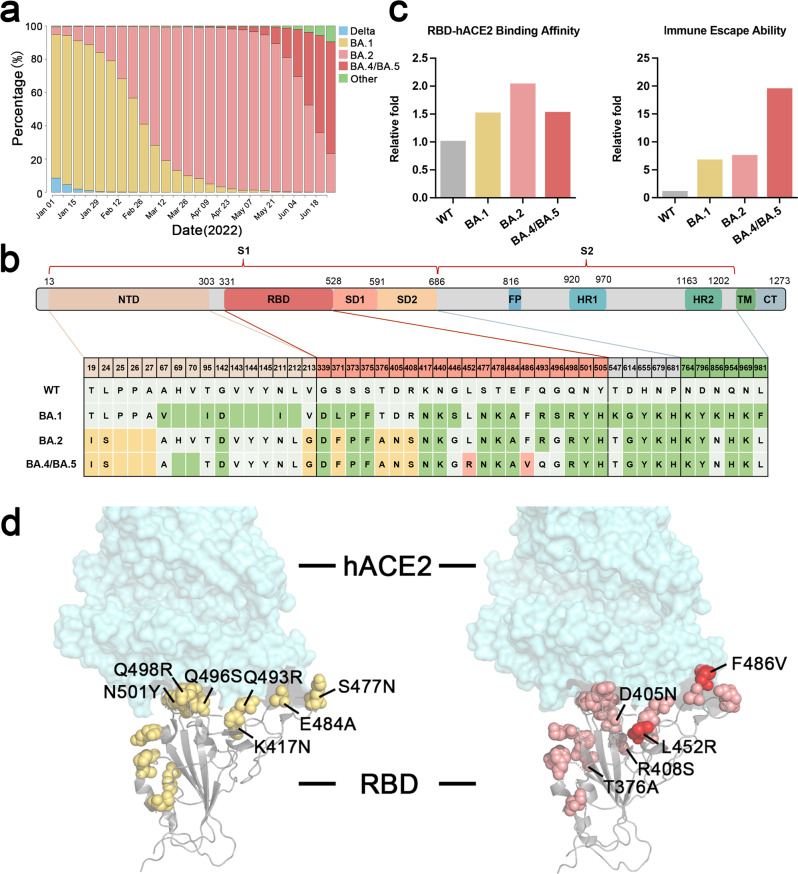
Comparison of prevalence percentage, structure and mutations, binding affinity, and immune escape capacity of Omicron subvariants BA.1, BA.2, and BA.4/5. **a** Prevalence percentage of Omicron subvariants since January 1, 2022 (Variant distribution data are available in https://covidcg.org/). **b** The mutations in the spike protein of Omicron subvariants BA.1, BA.2, and BA.4/BA.5, which are marked in different colors (green for new mutations in BA.1, yellow for new mutations in BA.2, pink for new mutations in BA.4/BA.5). **c** Comparison of RBD-hACE2 relative binding affinity and immune escape capacity among Wildtype (WT), BA.1, BA.2, and BA.4/BA.5. Data of binding affinity and immune escape capacity refer to the reported wet-bench data.^3,4^ **d** Structure and mutation annotation of RBD binding with hACE2. Yellow for mutations in Omicron BA.1, pink for mutations in BA.2, and pink and red for mutations in BA.4/BA.5

The emergent severe acute respiratory syndrome coronavirus 2 (SARS-CoV-2) variant Omicron (BA.x) and its sublineages with enhanced immune escape capacity have posed adverse impact and critical challenges to the efficacy of neutralizing antibody therapeutics and SARS-CoV-2 vaccines. Since the Omicron variant emerged in Africa in 2021, it rapidly becomes the dominant variant, replacing the former Delta and other variants around the world. Omicron BA.1 subvariant emergent in November 2021 was soon replaced by another Omicron subvariant BA.2 in early 2022. However, two subsequent emergent subvariants, BA.4/BA.5, found first in Africa with additional L452R and F486V mutations, competed with the former subvariant BA.2 with enhanced infectivity and antibody evasion ability^3,4^ (Fig. 1a, b).

Omicron variants carry large amount of mutations on the surface of the spike protein (>30 amino acid mutations), which have raised concerned of altering binding epitopes and causing escapes from the majority of existing SARS-CoV-2 therapeutical antibodies. Omicron BA.1 RBD forms extra interactions with ACE2 compared to wildtype strains (WT) due to the RBD mutations of S477N, Q493R, Q496S, Q498R, and N501Y as well as the mutations K417N and E484A compensating the loss of polar interactions (Fig. 1b).

According to the studies, the RBD-hACE2-binding affinity of Omicron BA.1 and BA.4/BA.5 is about 1.5-fold higher than WT, while BA.2 is about twofold higher than WT.^1–4^ (Fig.1c). With regard to the spike trimer, Omicronbound ACE2 with an increased affinity of about 6.2-folds (BA.1) and 11.1-folds (BA.2) compared with the WT spike trimer.^1,2^ BA.2 spike trimer bound ACE2 with an increased affinity of about 1.8-folds compared with BA.1. The unusual RBD-RBD interaction mechanism revealed that RBD in the Omicron spike trimer was stabilized in open-up conformation, which contributed to the higher affinity of Omicron variants. This may play an important role in its higher infectivity compared with former variants.^1^ Although the RBD of BA.1 spike trimer bound only one hACE2, Omicron BA.2 spike trimer could bind at least two or three hACE2, which was observed by cryo-EM structural analysis and indicated a stronger hACE2-binding capacity of the BA.2 spike trimer.^2^

In the study published in *Cell Research*, the high binding efficiency between Omicron spike trimer and mouse ACE2 was found, compared to WT spike trimer from strain Wuhan-Hu-1.^2^ However, both Omicron and WT spike trimers keep high binding efficiency with cat ACE2. These data suggest potential zoonotic events from Omicron variants and transmissible risks between infected human and animals.

Most Omicron mutations were found on the surface of the spike protein, many of which are the known epitopes targeted by the therapeutic antibodies. There are 15–17 mutations emergent in the Omicron RBD, which contain the receptor-binding sites and the epitopes for the major antibodies induced by infections or vaccinations. Previous studies have raised the concern of enhanced immune escape capacity from vaccinated sera and antibodies against Omicron variants^3,4^ (Fig.1c, d).

The RBD of newly emerged BA.4 and BA.5 subvariants encompasses the altered epitopes causing therapeutic antibody escape, e.g., Bril-196 of class 1, REGN10933 and LYCoV555 of Class 2, and REGN10987 and Bril-198 of Class 3.^1,3^ A broad-spectrum therapeutic antibody, JMB2002 which could neutralize diverse variants including Alpha, Beta, and Gamma, has completed the clinical trial of Phase I. It could also neutralize Omicron BA.1 and BA.2. JMB2002 Fab bound the Omicron spike trimer with increased affinity of BA.1 (K_D_ = 3.2 ± 3.0 nM) and BA.2 (approximately 2.6 nM) compared with the WT spike trimer (K_D_ = 12.2 ± 11.6 nM). Meanwhile, JMB2002 IgG bound the Omicron BA.1 (KD = 0.4 ± 0.1 nM) and BA.2 spike trimers (approximately 0.3 nM) more tightly than the WT (KD = 0.5 ± 0.3 nM). In pseudovirus neutralization assays, JMB2002 has equal inhibition efficacy in blocking the Omicron pseudovirus infection of human ACE2-expressing cells. The half-maximal inhibition concentration (IC50) was 0.2 μg/mL.

However, the L452R mutation in the Delta and BA.4/5 variants is located at the binding epitope position of JMB2002. This L452R mutation would block the Y102 binding of the heavy chain of JMB2002 Fab. Newly emerged BA.4 and BA.5 carrying L452R mutation and BA.2.12.1 subtype harboring L452Q mutation may evade JMB2002 Fab binding. The immune escape capacity of Omicron BA.4/BA.5 is about 20-fold higher than WT strain, and even threefold higher than BA.1 and BA.2^3,4^ (Fig. 1c). This has raised concern about reinfection and breakthrough infection among the recovered or vaccinated population. Surprisingly, the recurring L452R mutation has been identified earlier in some BA.1 and recombinant variants (“Deltacron”-like Variants).^5^ These diverse epitopes from continuously evolving variants suggest necessary cocktail antibody and vaccine antigen regimens to cover the altered epitopes.

In summary, the findings from Yin, Xu, and their colleagues provide the structural analysis of the Omicron BA.1 and BA.2 spike trimers and an effective anti-Omicron BA.1 and BA.2 antibody (JMB2002) along with its binding mechanism, which revealed the interaction between the Omicron spike trimer and hACE2, and discovered unusual RBD-RBD interaction mechanism, and suggested possible mouse origin of Omicron variant. The studies provide the mechanistic basis for the therapeutic antibody and vaccine development against SARS-CoV-2 and its continuously evolving variants. The composite cocktail antigens or antibodies targeting the diverse variant epitopes may contribute to the vaccine and drug development and tackle the challenges of continuously evolving Omicron variants.
